# Supplementary figures and images for: Nitrogen starvation leads to TOR kinase-mediated downregulation of fatty acid synthesis in the algae Chlorella sorokiniana and Chlamydomonas reinhardtii
Source: BMC Plant Biol. 2024 Aug 6;24:753. doi: 10.1186/s12870-024-05408-7 (PMC11302099; doi:10.1186/s12870-024-05408-7)

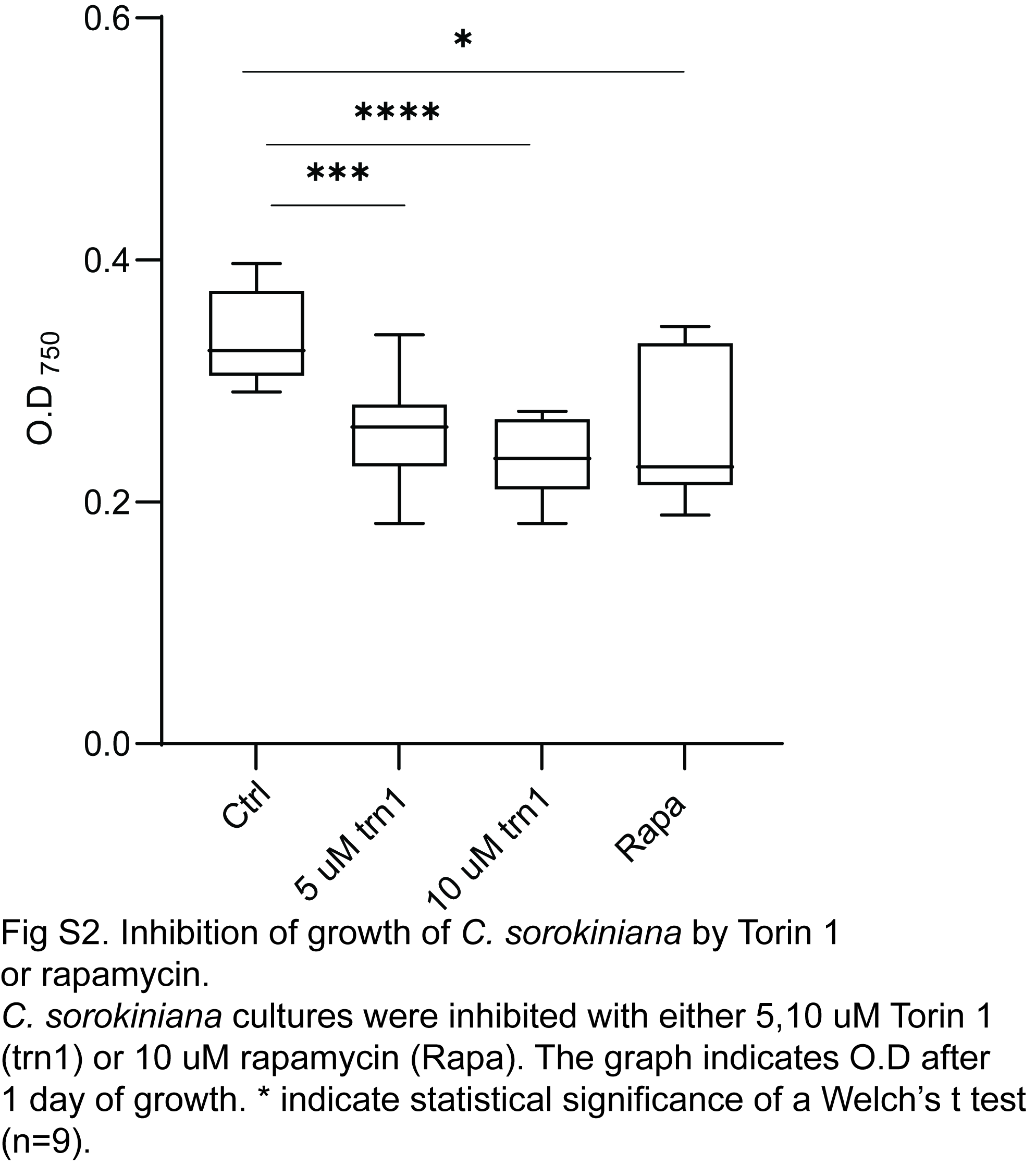

Supplement: Supplementary file 6 — Supplementary Material 6. TOR Supplement Fig 2. Inhibition of growth of C. sorokiniana by rapamycin and Torin 1. [file 12870_2024_5408_MOESM6_ESM.tif]

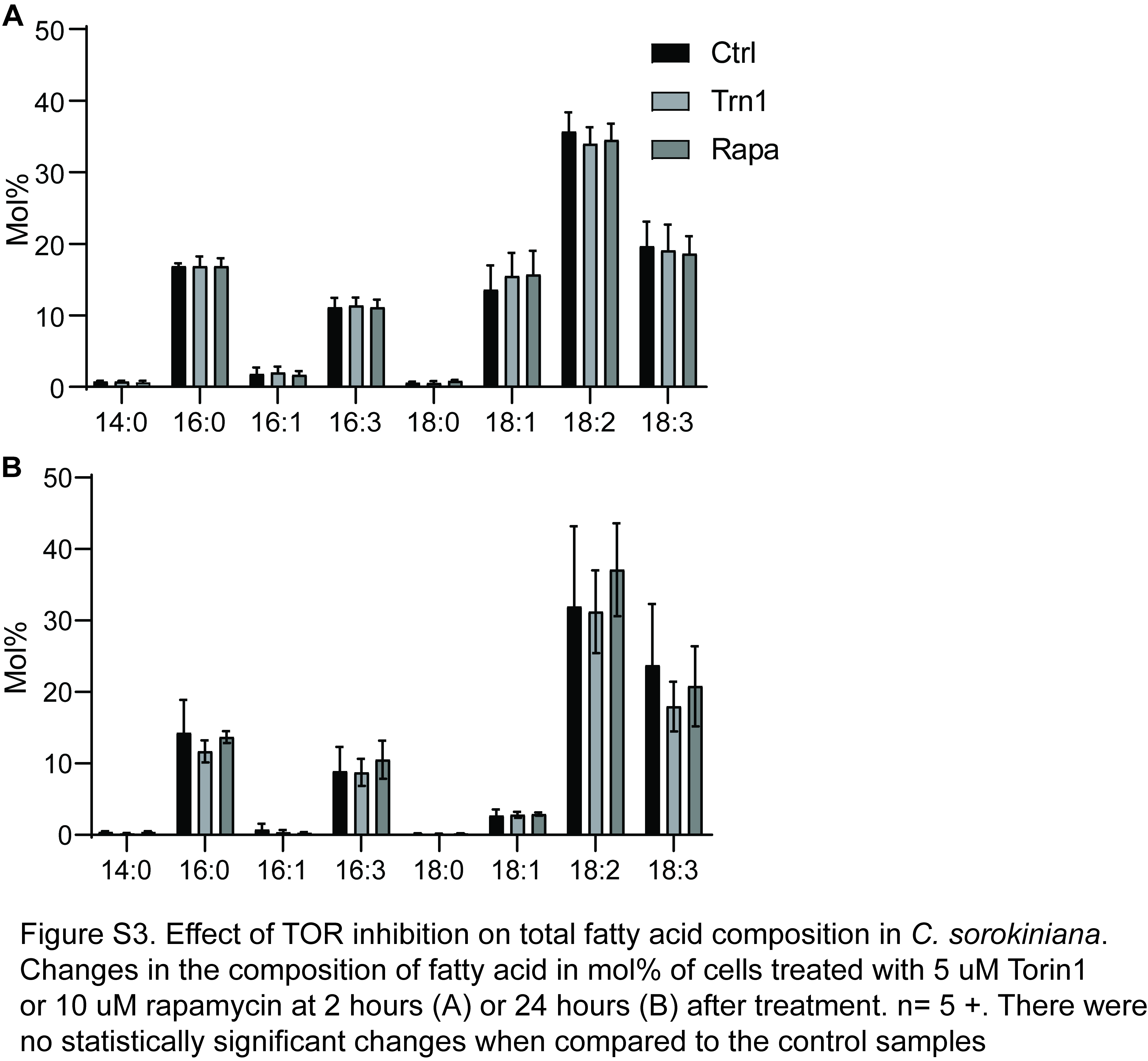

Supplement: Supplementary file 7 — Supplementary Material 7. TOR Supplement Fig 3. Fatty acid composition of C. sorokiniana cells treated with Torin 1 or rapamycin at 2 and 24 hours after treatment. [file 12870_2024_5408_MOESM7_ESM.tif]
